# Alcohol intake and ovarian cancer risk: a pooled analysis of 10 cohort studies

**DOI:** 10.1038/sj.bjc.6603020

**Published:** 2006-02-21

**Authors:** J M Genkinger, D J Hunter, D Spiegelman, K E Anderson, J E Buring, J L Freudenheim, R A Goldbohm, L Harnack, S E Hankinson, S C Larsson, M Leitzmann, M L McCullough, J Marshall, A B Miller, C Rodriguez, T E Rohan, A Schatzkin, L J Schouten, A Wolk, S M Zhang, S A Smith-Warner

**Affiliations:** 1 Department of Nutrition, Harvard School of Public Health, Boston, MA, USA; 2 Department of Epidemiology, Harvard School of Public Health, Boston, MA, USA; 3 Channing Laboratory, Department of Medicine, Brigham and Women's Hospital and Harvard Medical School, Boston, MA, USA; 4 Department of Biostatistics, Harvard School of Public Health, Boston, MA, USA; 5 Division of Epidemiology, School of Public Health, University of Minnesota, Minneapolis, MN, USA; 6 Division of Preventive Medicine, Department of Medicine, Brigham and Women's Hospital and Harvard Medical School, Boston, MA, USA; 7 Department of Social and Preventive Medicine, University at Buffalo, State University of New York, Buffalo, NY, USA; 8 Department of Food and Chemical Risk Analysis, TNO Quality of Life, Zeist, The Netherlands; 9 Division of Nutritional Epidemiology, National Institute of Environmental Medicine, Karolinska Institute, Stockholm, Sweden; 10 Division of Cancer Epidemiology and Genetics, National Cancer Institute, NIH, DHHS, Bethesda, MD, USA; 11 Epidemiology and Surveillance Research, American Cancer Society, Atlanta, GA, USA; 12 Department of Public Health Sciences, Faculty of Medicine, University of Toronto, Toronto, Ontario, Canada; 13 Department of Epidemiology and Population Health, Albert Einstein College of Medicine, Bronx, NY, USA; 14 Department of Epidemiology, NUTRIM, Maastricht University, Maastricht, The Netherlands

**Keywords:** alcohol, ovarian cancer, pooled analysis

## Abstract

Alcohol has been hypothesized to promote ovarian carcinogenesis by its potential to increase circulating levels of estrogen and other hormones; through its oxidation byproduct, acetaldehyde, which may act as a cocarcinogen; and by depletion of folate and other nutrients. Case–control and cohort studies have reported conflicting results relating alcohol intake to ovarian cancer risk. We conducted a pooled analysis of the primary data from ten prospective cohort studies. The analysis included 529 638 women among whom 2001 incident epithelial ovarian cases were documented. After study-specific relative risks (RR) and 95% confidence intervals (CI) were calculated by Cox proportional hazards models, and then were pooled using a random effects model; no associations were observed for intakes of total alcohol (pooled multivariate RR=1.12, 95% CI 0.86–1.44 comparing ⩾30 to 0 g day^−1^ of alcohol) or alcohol from wine, beer or spirits and ovarian cancer risk. The association with alcohol consumption was not modified by oral contraceptive use, hormone replacement therapy, parity, menopausal status, folate intake, body mass index, or smoking. Associations for endometrioid, mucinous, and serous ovarian cancer were similar to the overall findings. This pooled analysis does not support an association between moderate alcohol intake and ovarian cancer risk.

Alcohol intake has been associated with a higher risk of cancers of the upper alimentary tract, liver, large intestine, and breast (Poschl and Seitz, 2004). Alcohol has been theorized to promote carcinogenesis by its potential to increase circulating levels of oestrogen and other hormones; through its oxidation byproduct, acetaldehyde, which may act as a cocarcinogen; by induction of cytochrome P450 enzymes which are involved in the activation of liver carcinogens; and by depletion of folate (Poschl and Seitz, 2004). In contrast, alcohol has also been hypothesized to prevent ovarian carcinogenesis by decreasing follicle stimulating hormone (Gavaler and van Thiel, 1992), luteinizing hormone (Gavaler and van Thiel, 1992) and gonadotropins (Verkasalo *et al*, 2001) levels, all of which have been hypothesized to increase ovarian cancer risk (Cramer and Welch, 1983).

Several epidemiologic studies have examined the association between alcohol consumption and ovarian cancer risk (McCann *et al*, 2003; Modugno *et al*, 2003; Kelemen *et al*, 2004; Larsson *et al*, 2004; Riman *et al*, 2004; Webb *et al*, 2004). A recent meta-analysis reported a 30% lower risk of ovarian cancer risk with higher intakes of alcohol (Webb *et al*, 2004) for population based case–control and cohort studies, but found no association for hospital-based case–control studies. Overall, the results from cohort studies of women with alcoholism have been nonstatistically significant, with suggestions of positive and inverse associations between alcohol intake and ovarian cancer risk (Webb *et al*, 2004). One of two case–control studies has found that alcohol intake was positively associated with risk of mucinous tumours (Kuper *et al*, 2000; Modugno *et al*, 2003).

Owing to the potential for recall bias in case–control studies and the small number of cases in most cohort studies, we investigated the associations between alcohol intake and ovarian cancer risk in a pooled analysis of several prospective studies. As the effect of alcohol may vary by potential ovarian cancer risk factors, we also considered whether the association differed by hormonal, environmental and nutritional factors. We also examined associations separately for endometrioid, mucinous, and serous ovarian cancers.

## MATERIALS AND METHODS

### Population

A pooled analysis of the primary data from 10 prospective cohort studies (Bandera *et al*, 1997; Rockhill *et al*, 1998; Bertone *et al*, 2002; Calle *et al*, 2002; Lacey *et al*, 2002; Terry *et al*, 2003; Kelemen *et al*, 2004; Larsson *et al*, 2004; Lin *et al*, 2004; Schouten *et al*, 2004) was conducted in The Pooling Project of Prospective Studies of Diet and Cancer (Table 1). To be included in this analysis, each study needed a minimum of 50 incident ovarian cancer cases, an assessment of usual diet, including alcohol intake, and validation of the dietary assessment tool or a closely related instrument.

Two studies, the Canadian National Breast Screening Study and Netherlands Cohort Study, were analysed as case-cohort studies because the investigators have processed questionnaires for newly diagnosed cases and a random sample of noncases to represent the person-time of the entire study population. The follow-up of the Nurses' Health Study was divided into two sections where NHS (a) followed individuals from the completion of the 1980 food frequency questionnaire (FFQ) to 1986, and NHS (b) followed those individuals still at risk from the completion of the 1986 FFQ (a more comprehensive FFQ compared to the 1980 questionnaire) to 2002. The methods for the Pooling Project have been described in detail elsewhere (Smith-Warner *et al*, in press).

### Exclusions

In addition to applying the exclusions that each study had predefined for their cohort, we excluded individuals if they had a prior cancer diagnosis other than nonmelanoma skin cancer at baseline, had a bilateral oophorectomy prior to baseline, had log_e_-transformed energy intakes beyond 3 s.d. from the study-specific log_e_-transformed mean energy intake of their respective population, or had missing alcohol intake data. The New York State Cohort (Bandera *et al*, 1997) did not obtain information on oophorectomy at baseline, and thus we were not able to apply that exclusion there.

### Exposure assessment

Usual dietary intake was estimated at baseline from study-specific FFQs. All studies, except the New York State Cohort, measured alcohol intake from wine, beer, and spirits (see Table 1). In these studies, daily alcohol intake in grams was calculated for each beverage based on the reported frequency of consumption, the alcohol content of the beverage, and the average quantity consumed. The alcohol intake from each specific beverage was summed to estimate total alcohol intake. Intakes of other nutrients were estimated using a similar approach. In the New York State Cohort, the ‘regression weight’ method was used to estimate nutrient and alcohol values (Bandera *et al*, 1997). Correlations between energy-adjusted alcohol intake measured from the study-specific FFQ or a closely related instrument and multiple 24 h recalls or food records generally exceeded 0.77 for total alcohol intake (Giovannucci *et al*, 1991; Munger *et al*, 1992; Goldbohm *et al*, 1994; Flagg *et al*, 2000; Wolk *et al*, personal communication).

Information on nondietary factors was collected on the baseline self-administered questionnaires within each study. Most studies obtained information on multiple reproductive factors, body mass index, smoking status, and physical activity.

### Outcome assessment

Participants were followed from the date of the baseline questionnaire until date of ovarian cancer diagnosis, date of death, date the participant moved out of the study area (if applicable), or end of follow-up, whichever came first. Invasive epithelial ovarian cancer, defined by ICD-9 code 183.0 or ICD-10 code C56, was ascertained by self-report with subsequent medical record review, cancer registry linkage, or both. Some studies additionally obtained information from death registries (Smith-Warner *et al*, in press). Borderline and nonepithelial ovarian cancer cases, as determined by International Classification of Diseases for Oncology morphology codes (Percy *et al*, 1990) or the histological information supplied by individual studies, were not included as cases.

### Statistical analysis

Alcohol intake was modelled categorically within each individual study using identical absolute cutpoints. Studies were excluded from the analysis if they did not measure intake of that specific beverage or if that beverage was not consumed in that population.

Relative risks (RR) and 95% confidence intervals (CI) were calculated by Cox proportional hazards models (Cox, 1972) for each study. The models included stratification by age (years) at baseline and the calendar year at start of follow-up, and treated follow-up time (years) as the time scale. Multivariate relative risks were adjusted for age at menarche, menopausal status at baseline, oral contraceptive use, hormone replacement therapy use among postmenopausal women, parity, body mass index, smoking status, physical activity, and energy intake, modelled identically across studies. An indicator variable for missing values was also generated within a study for each covariate, if applicable (Smith-Warner *et al*, in press). To test whether there was a linear trend in the risk of disease with increasing alcohol intake, a continuous variable with values corresponding to the median value for each exposure category was included in the model; the coefficient for that variable was evaluated using the Wald test. SAS software (Allison, 1995) was used for the cohort analyses, and Epicure software (1993) was used for the case-cohort analyses.

Study-specific relative risks were pooled using a random effects model (DerSimonian and Laird, 1986). The study-specific relative risks were weighted by the inverse of the sum of their variance and the estimated between-studies variance component. Between-studies heterogeneity was evaluated using the Q statistic (Cochran, 1954; DerSimonian and Laird, 1986).

We also tested whether the associations for alcohol from beer, wine, and liquor and ovarian cancer risk differed by using a contrast test for the pooled estimates for each beverage and their covariance matrix from the random-effects model. The test statistic followed approximately a *χ*^2^ distribution with 2 degrees of freedom (Anderson, 1984). For each study, we corrected the relative risk for total alcohol intake for measurement error using the regression coefficients between alcohol intakes estimated by the FFQs and reference methods (Rosner *et al*, 1989, 1990).

We also evaluated whether alcohol intake was linearly associated with ovarian cancer risk by comparing nonparametric regression curves using restricted cubic splines to the linear model using the likelihood ratio test, and by visual inspection of the restricted cubic spline graphs (Smith, 1979; Durrleman and Simon, 1989). The studies were combined into a single data set, and analysed as above, additionally stratified by study. Owing to the small numbers of individuals consuming high amounts of alcohol, this analysis was limited to women consuming <60 g day^−1^ of alcohol.

Further analyses were conducted to examine whether the association between alcohol consumption and ovarian cancer risk varied by hormonal, environmental and nutritional factors. As most studies collected information at baseline only, for analyses evaluating whether menopausal status modified the association between alcohol consumption and ovarian cancer risk, we assigned menopausal status at follow-up in each study using a previously described algorithm (Smith-Warner *et al*, 1998). For each factor, an interaction term between the level of each factor and alcohol intake expressed as a continuous variable was included in the model. Participants with missing values of the modifying factor were excluded from these analyses. Separate analyses were also conducted for endometrioid, mucinous, and serous subtypes among those studies having more than 10 cases of the specific subtype. Subtype analyses were conducted among these three histologies since they are the three most common, representing 68% of all ovarian cancer cases in our population. We tested whether results differed across the subtypes using a contrast test (Anderson, 1984).

## RESULTS

The total study population consisted of 529 638 women among whom 2001 developed invasive epithelial ovarian cancer over 4.6 million person years (Table 1). The percentage of alcohol drinkers in each study ranged from 45 to 78%. Median total alcohol intake among drinkers was low and ranged from 1.9 to 6.6 g day^−1^.

Alcohol intake was not associated with ovarian cancer risk (pooled age-adjusted RR=1.11, 95% CI: 0.87–1.43 and multivariate RR=1.12, 95% CI: 0.86–1.44) comparing ⩾30 to 0 g day^−1^ (Table 2). There was no significant between-studies heterogeneity (*P*-value, test for between-studies heterogeneity >0.50). Likewise, no association was observed for alcohol from individual beverages and ovarian cancer risk (Table 3). Results were similar after excluding nondrinkers (data not shown).

The nonparametric regression curve also showed no association with alcohol intake and ovarian cancer risk (test for linearity, *P*>0.05) (data not shown). Among the five studies that assessed alcohol intake in validation studies (Giovannucci *et al*, 1991; Munger *et al*, 1992; Goldbohm *et al*, 1994; Flagg *et al*, 2000; Wolk *et al*, personal communication), the pooled age and energy-adjusted relative risk for an increment of 15 g day^−1^ of alcohol (equivalent to approximately one serving of alcohol) changed from 1.01 (95% CI: 0.91–1.11) to 1.04 (95% CI: 0.92–1.18; *P*-value, test for between studies heterogeneity=0.91) after correction for measurement error in the assessment of alcohol consumption. For all cohorts, the pooled multivariate RR for this increment was 1.01 (95% CI: 0.93–1.11). In analyses that simultaneously adjusted for intakes of alcohol from wine, beer, and spirits as continuous variables (increment=15 g day^−1^), we found no association of alcohol from wine (pooled multivariate RR=1.07, 95% CI: 0.95–1.21), beer (pooled multivariate RR=1.02, 95% CI: 0.84–1.24) and spirits (pooled multivariate RR=1.03, 95% CI: 0.93–1.14) with ovarian cancer risk (*P*-value for the test of difference=0.83). These results were similar to those not mutually adjusted for intake of alcohol from other beverages.

Risk estimates for total alcohol intake were similar for endometrioid (*N*=260, pooled multivariate RR=1.05, 95% CI: 0.87–1.26), mucinous (*N*=121, pooled multivariate RR=1.06, 95% CI: 0.84–1.34) and serous (*N*=981, pooled multivariate RR=1.07, 95% CI: 0.98–1.17) ovarian cancers (*P*-value for difference by histological type=0.98).

The association between total alcohol intake and ovarian cancer risk was not modified by hormonal, environmental and nutritional factors (Table 4). The relative risks for total alcohol did not differ when the cases were stratified into two groups defined by age at diagnosis (data not shown). Sensitivity analyses in which we limited analyses to the first 6 years of follow-up compared to six or more years of follow-up showed similar results to the overall result (data not shown).

## DISCUSSION

In this pooled analysis of prospective studies, no statistically significant associations were observed for alcohol intake overall, or from specific beverages, with ovarian cancer risk. In contrast to the inverse results published in a recent meta-analysis (Webb *et al*, 2004) of five population-based case–control and two cohort studies, our null results were similar to those reported in three recently published population-based case–control studies (McCann *et al*, 2003; Modugno *et al*, 2003; Riman *et al*, 2004) and in the meta-analysis (Webb *et al*, 2004) of seven hospital-based case–control studies. Unlike two case–control studies (Goodman and Tung, 2003; Webb *et al*, 2004), we did not observe an inverse association between wine intake and ovarian cancer risk.

Two case–control studies have examined alcohol intake in relation to serous, mucinous, and endometrioid ovarian cancers risk separately (Kuper *et al*, 2000; Modugno *et al*, 2003). One study saw a higher risk of mucinous ovarian cancer with higher alcohol intake (Modugno *et al*, 2003). In contrast, we found no difference in the associations between alcohol consumption and the risk of specific histological types of ovarian cancer. Although some misclassification is likely to have occurred in our analyses with regard to classifying cases according to histology, the distribution of cases according to the three main histological types in our study was similar to the percent distribution observed in cancer registries and other studies for the main histological subtypes of ovarian cancer (Scully, 1977; Chen *et al*, 2003).

As our analyses were conducted using only a baseline FFQ covering recent intake, we were unable to assess changes in intakes of alcohol and other nutrients over time, nor were we able to evaluate lifetime drinking patterns. Only a few studies have addressed these additional ways to examine alcohol drinking habits in relation to ovarian cancer risks and the results have been heterogenous (Tzonou *et al*, 1984; Goodman and Tung, 2003). Additionally, because we measured adult alcohol intake, we may not have captured the relevant exposure time for ovarian carcinogenesis.

In the studies comprising this pooled analysis, diet was measured prior to diagnosis of ovarian cancer; thus, a cancer diagnosis would not have influenced the reporting of alcohol intake. The strength of our study was our ability to correct for measurement error in alcohol intake. The measurement error corrected risk estimates were similar to the uncorrected results which was expected because the assessments of alcohol intake from the study-specific questionnaires and multiple 24 h recalls or food records have been shown to be highly correlated (Giovannucci *et al*, 1991; Munger *et al*, 1992; Goldbohm *et al*, 1994; Flagg *et al*, 2000; Wolk *et al*, personal communication). In addition, we have previously observed a modest positive association between alcohol intake and breast (Smith-Warner *et al*, 1998) and colorectal cancer (Cho *et al*, 2004) risk within the Pooling Project suggesting that misclassification of alcohol intake is not likely to obscure a modest association, although we cannot rule out the possibility of a small effect.

Although our categorization of covariates was predetermined according to how each study assessed the covariates on their questionnaires, one advantage of our study was that we were able to control for covariates uniformly and classify the main exposures similarly, thereby lessening a potential source of heterogeneity across studies. Within our models, we adjusted for most of the important known ovarian cancer risk factors if they were measured in a study. In studies that measured all of the covariates that were included in our multivariate models, results from the age-adjusted and multivariate models were similar suggesting that confounding was minimal. Owing to the inclusion of ten cohort studies, we had far greater statistical power than any individual cohort study to examine whether associations differed for histological subtypes or population subgroups.

In summary, we found no association between intakes of alcohol and alcohol from specific beverages during adulthood and ovarian cancer risk in this pooled analysis. Similarly, we observed no association between alcohol consumption and ovarian cancer risk by histology. Associations with alcohol intake were not modified by risk or preventive factors for ovarian cancer. Thus, this pooled analysis does not provide support for an association between moderate alcohol intake and ovarian cancer risk.

## Figures and Tables

**Table 1:** Daily median intakes (interquartile range) of alcohol (g day^−1^) among drinkers by cohort study in the ovarian cancer analyses in the Pooling Project of Prospective Studies of Diet and Cancer

						**Median intake (interquartile range) of alcohol (g day^−1^) among drinkers^d^**			
**Cohort^a^**	**Follow-up years**	**Baseline cohort size^b^**	**Number of cases^b^**	**Baseline age range, years**	**Percent of drinkers**	**Total alcohol intake**	**Alcohol intake from wine**	**Alcohol intake from beer**	**Alcohol intake from spirits**
BCDDP	1987–1999	32 885	142	40–93	52	3.1 (0.9–10.4)	1.4 (0.4–4.6)	0.9 (0.3–1.9)	2.2 (0.5–6.5)
CNBSS^c^	1980–2000	49 613	223	40–59	77	6.6 (2.4–14.9)	3.1 (1.0–7.1)	1.7 (0.9–3.7)	2.3 (1.1–6.8)
CPS II	1992–2001	60 796	278	50–74	53	4.3 (1.1–11.3)	1.4 (0.7–5.8)	0.9 (0.9–2.8)	2.1 (1.0–11.0)
IWHS	1986–2001	28 486	208	55–69	45	3.6 (1.7–11.2)	1.7 (0.9–3.0)	1.8 (1.1–5.7)	2.1 (1.2–6.5)
NLCS^c^	1986–1995	62 412	195	55–69	68	4.1 (1.4–12.0)	3.2 (1.2–9.6)	1.1 (0.5–2.8)	4.6 (1.1–12.1)
NYSC^e^	1980–1987	22 550	77	50–93	78	1.9 (0.5–9.5)	—	—	—
NHS(a)	1980–1986	80 195	120	34–59	68	5.5 (1.8–12.1)	1.5 (0.9–4.7)	1.8 (1.0–5.5)	2.0 (1.1–6.0)
NHS(b)	1986–2002	59 538	315	40–65	65	5.0 (1.9–12.4)	1.8 (0.9–5.6)	1.8 (1.0–5.5)	2.0 (1.1–6.0)
NHS II	1991–2002	91 502	52	27–44	57	2.8 (1.5–6.6)	1.5 (0.9–3.0)	2.0 (1.1–4.9)	1.2 (1.2–2.1)
SMC^c^	1987–2003	61 103	287	40–74	67	2.8 (1.5–4.6)	1.8 (1.6–3.3)	0.9 (0.4–1.8)	1.0 (0.9–1.0)
WHS	1993–2004	32 466	104	45–89	60	3.8 (1.8–10.2)	1.7 (0.9–5.5)	1.8 (0.9–3.4)	2.1 (1.2–6.5)

a BCDDP=Breast Cancer Detection Demonstration Project Follow-up Study, CNBSS=Canadian National Breast Screening Study, CPS II=Cancer Prevention Study II Nutrition Cohort, IWHS=Iowa Women's Health Study, NLCS=Netherlands Cohort Study, NYSC=New York State Cohort, NHS(a)=Nurses' Health Study (part a), NHS(b)=Nurses' Health Study (part b), NHS II=Nurses' Health Study II, SMC=Swedish Mammography Cohort, WHS=Women's Health Study.

b Baseline cohort size and number of cases determined after specific exclusions (ie, prior cancer diagnosis other than nonmelanoma skin cancer at baseline, bilateral oophorectomy prior to baseline, or log_e_-transformed energy intakes beyond three standard deviations from the study-specific log_e_-transformed mean energy intake of the population).

c CNBSS and NLCS are analyzed as case-cohort studies so the baseline cohort size does not reflect the above exclusions.

d Median intakes are g day^−1^ for alcohol and alcohol from specific beverages among drinkers of that beverage.

e NYSC did not measure consumption of alcohol from specific beverages.

**Table 2:** Study-specific and pooled multivariate^a^ adjusted relative risks and 95% confidence intervals for ovarian cancer according to intake of total alcohol

	**Categories of alcohol intake (g day^−1^)**						
**Study**	**0**	**1**–**4.9**	**5**–**14.9**	**15**–**29.9**	**⩾30**	***P*_Heterogeneity_ c**	***P*_trend_ d**
Breast Cancer Detection Demonstration Project Follow-up Study	1.00 (Ref)	1.25 (0.84–1.87)	1.39 (0.82–2.35)	1.70 (0.87–3.33)	2.21 (0.92–5.31)		0.04
Canadian National Breast Screening Study	1.00 (Ref)	0.90 (0.61–1.33)	1.21 (0.81–1.80)	1.10 (0.67–1.81)	1.27 (0.71–2.25)		0.24
Cancer Prevention Study II Nutrition Cohort	1.00 (Ref)	1.07 (0.81–1.41)	0.85 (0.59–1.22)	0.82 (0.43–1.53)	0.79 (0.39–1.58)		0.25
Iowa Women's Health Study	1.00 (Ref)	1.28 (0.94–1.75)	0.87 (0.53–1.41)	0.71 (0.31–1.64)	0.75 (0.30–1.88)		0.24
Netherlands Cohort Study	1.00 (Ref)	1.06 (0.73–1.52)	1.07 (0.67–1.70)	0.77 (0.39–1.53)	1.79 (0.83–3.85)		0.50
New York State Cohort	1.00 (Ref)	0.98 (0.58–1.65)	0.28 (0.08–0.96)	0.41 (0.09–1.81)	0.53 (0.07–4.02)		0.05
Nurses' Health Study(a)	1.00 (Ref)	0.90 (0.58–1.40)	0.82 (0.49–1.36)	0.92 (0.44–1.91)	0.78 (0.32–1.87)		0.57
Nurses' Health Study(b)	1.00 (Ref)	1.13 (0.86–1.50)	0.85 (0.60–1.20)	1.52 (1.01–2.29)	0.87 (0.49–1.56)		0.96
Nurses' Health Study II	1.00 (Ref)	0.94 (0.52–1.72)	0.73 (0.29–1.83)	0.60 (0.08–4.50)	1.59 (0.21–12.1)		0.84
Swedish Mammography Cohort^b^	1.00 (Ref)	1.04 (0.80–1.35)	1.04 (0.68–1.59)	1.19 (0.29–4.85)	—		0.79
Women's Health Study	1.00 (Ref)	0.78 (0.48–1.27)	1.10 (0.65–1.87)	1.04 (0.43–2.49)	1.66 (0.64–4.32)		0.21
							
Cases	754	729	320	122	76		
Pooled	1.00 (Ref)	1.06 (0.95–1.18)	0.96 (0.84–1.11)	1.09 (0.89–1.35)	1.12 (0.86–1.44)	0.50	0.72

a Multivariate relative risks were adjusted for calendar year age at menarche (<13, 13, >13 years), menopausal status at baseline (premenopausal, postmenopausal, dubious), oral contraceptive use (ever, never), hormone replacement therapy use among postmenopausal women (never, past, current), parity (0, 1, 2, >2,), body mass index (<23, 23-<25, 25-<30, ⩾30 kg m^−2^), smoking status (never, past, current), physical activity (low, medium, high), and energy intake (continuous).

b Swedish Mammography Cohort was not included in the category ⩾30 g of alcohol since this study had no cases in that category. The participants who were not cases who would have been in this highest category were included in the next highest category (15–29.9 gday^−1^).

c *P-*value, test for between-studies heterogeneity is for the ⩾30 g day^−1^ category.

d *P-*value, test for trend.

**Table 3:** Pooled age and multivariate^a^^,^^b^ adjusted relative risks (RR) and 95% confidence intervals (CI) for ovarian cancer according to intake of alcohol from specific beverages

	**Categories of alcohol intake (g day^−1^)**					
**Range**	**0**	**1**–**4.9**	**5**–**14.9**	**⩾15**	***P*_Heterogeneity_ f**	***P*_trend_ g**
*Wine* c						
Cases	965	719	174	66		
Age RR (95% CI)	1.00 (Ref)	1.04 (0.94–1.15)	1.05 (0.89–1.25)	1.21 (0.92–1.60)	0.77	0.35
MV RR (95% CI)	1.00 (Ref)	1.03 (0.93–1.14)	1.03 (0.86–1.23)	1.18 (0.89–1.57)	0.80	0.49
						
*Beer* d						
Cases	1462	396	48	18		
Age RR (95% CI)	1.00 (Ref)	1.11 (0.98–1.25)	0.76 (0.57–1.02)	1.40 (0.87–2.25)	0.82	0.76
MV RR (95% CI)	1.00 (Ref)	1.09 (0.97–1.24)	0.75 (0.56–1.01)	1.38 (0.85–2.25)	0.87	0.78
						
*Spirits* e						
Cases	1335	380	139	70		
Age RR (95% CI)	1.00 (Ref)	1.01 (0.89–1.15)	1.03 (0.78–1.37)	1.17 (0.85–1.61)	0.15	0.59
MV RR (95% CI)	1.00 (Ref)	1.01 (0.89–1.15)	1.02 (0.75–1.38)	1.16 (0.85–1.57)	0.21	0.68

a Age adjusted relative risks were adjusted for age and calendar year. Multivariate relative risks were adjusted for the same covariates as the multivariate model in Table 2. In these analyses, alcohol from wine, beer and spirits were not mutually adjusted.

b New York State Cohort was not included in these analyses because they did not measure consumption of alcohol from specific beverages.

c Nurses' Health Study II was not included in the 5–14.9 and ⩾15 g day^−1^ categories of alcohol from wine since this study had no cases in that category.

d Nurses' Health Study II and Swedish Mammography Cohort were not included in the ⩾15 g day^−1^ category of alcohol from beer since these studies had no cases in that category. The participants who were not cases who would have been in this highest category were included in the next highest category (5–14.9 g day^−1^).

e Swedish Mammography Cohort was not included in the ⩾15 g day^−1^ category of alcohol from spirits since this study had no cases in that category.

f *P-*value, test for between-studies heterogeneity is for ⩾15 gday^−1^ category.

g *P-*value, test for trend.

**Table 4:** Pooled multivariate relative risks^a^ and 95% confidence intervals for a 15 g day^−1^ increment in total alcohol intake by levels of other ovarian cancer risk factors, continuous model

**Factor**	**Cases**	**Relative risk (95% CI)**	***P*-value, test for interaction**
*Hormonal/reproductive*			
Parity (live birth)			
⩽1	518	1.02 (0.88–1.20)	
>1	1435	1.02 (0.94–1.12)	>0.99
Oral contraceptive use^b^			
Never	1124	1.02 (0.88–1.18)	
Ever	599	1.05 (0.96–1.16)	0.41
Hormone replacement therapy^c^^,^^d^			
Never	634	1.02 (0.84–1.23)	
Past	239	1.19 (0.99–1.44)	
Current	211	1.10 (0.90–1.35)	0.39
Menopausal status at diagnosis^e^			
Premenopausal^f^	140	0.72 (0.39–1.32)	
Postmenopausal^g^	1301	1.05 (0.94–1.16)	0.24
			
*Nutritional*			
Methionine intake (median)			
Low	990	1.02 (0.94–1.12)	
High	1011	1.04 (0.85–1.26)	0.34
Folate intake (median)^h^			
Low	743	0.97 (0.86–1.09)	
High	748	1.05 (0.89–1.24)	0.18
Multivitamin use^i^			
No	959	1.01 (0.90–1.13)	
Yes	513	1.01 (0.83–1.23)	0.56
			
*Other*			
Smoking status^j^			
Never	914	0.97 (0.84–1.13)	
Past	499	1.09 (0.96–1.23)	
Current^k^	285	1.05 (0.87–1.28)	0.52
Body mass index (kg m^−2^)			
⩽23	707	1.00 (0.90–1.11)	
23–<25	416	1.06 (0.93–1.21)	
25–<30	566	1.07 (0.93–1.23)	
⩾30	261	1.03 (0.80–1.33)	0.74

a Multivariate relative risks were adjusted for the same covariates as the multivariate model in Table 2. Within each model, the stratification variable was excluded from the model.

b New York State Cohort was excluded in this analysis because they did not measure oral contraceptive use on the baseline questionnaire.

c Canadian National Breast Screening Study, New York State Cohort, and Swedish Mammography Cohort were excluded from this analysis because they did not measure never, past or current hormone replacement therapy use at baseline.

d Netherlands Cohort Study and Nurses' Health Study(a) were excluded from the current hormone replacement therapy use analysis due to small case numbers (*n*<10).

e New York State Cohort was excluded from this analysis because they did not measure menopausal status on the baseline questionnaire.

f Breast Cancer Detection Demonstration Project Follow-up Study, Cancer Prevention Study II Nutrition Cohort, Iowa Women's Health Study, Netherlands Cohort Study, and Swedish Mammography Cohort were excluded from the premenopausal analysis due to small case numbers (*n*<10).

g Nurses' Health Study II was excluded from the postmenopausal analysis due to small case numbers (*n*<10).

h Canadian National Breast Screening Study and Swedish Mammography Cohort were excluded because they folate intake includes dietary and supplemental sources did not have the use of multivitamins and single supplement data available.

i Canadian National Breast Screening Study and Swedish Mammography Cohort were excluded because they did not have use of multivitamin data available.

j Swedish Mammography Cohort was excluded from this analysis because they did not measure smoking at baseline.

k Nurses Health Study II was excluded from this analysis due to small case numbers (*n*<10).
